# Restored and Enhanced Memory T Cell Immunity in Rheumatoid Arthritis After TNFα Blocker Treatment

**DOI:** 10.3389/fimmu.2019.00887

**Published:** 2019-04-24

**Authors:** Asma Khanniche, Ling Zhou, Bin Jiang, Jing Song, Yanhua Jin, Jian Yin, Shujun Wang, Ping Ji, Hao Shen, Ying Wang, Huji Xu

**Affiliations:** ^1^Shanghai Institute of Immunology, Shanghai Jiao Tong University School of Medicine, Shanghai, China; ^2^Shenzhen Institutes of Advanced Technology, Chinese Academy of Sciences, Shenzhen, China; ^3^Department of Rheumatology and Immunology, Shanghai Chang Zheng Hospital, Second Military Medical University, Shanghai, China; ^4^Department of Rheumatology, Renji Hospital, Shanghai, China; ^5^Department of Microbiology, Perelman School of Medicine, University of Pennsylvania, Philadelphia, PA, United States; ^6^Beijing Tsinghua Changgung Hospital, School of Clinical Medicine, Tsinghua University, Beijing, China; ^7^Peking-Tsinghua Center for Life Sciences, Tsinghua University, Beijing, China

**Keywords:** rheumatoid arthritis, memory T cells, antigen specific, TNFα inhibitor, immune regulation

## Abstract

TNFα inhibitors have shaped the landscape of rheumatoid arthritis (RA) therapy with high clinical efficiency. However, their impact on T cell recall responses is not well-elucidated. We aimed to analyze the immune profiles of memory T cells in RA patients undergoing TNFα inhibitor Golimumab (GM) treatment. Frequencies of peripheral T cell subsets and cytokine expression profiles in memory T cells (T_M_) upon PMA/Ionomycine stimulation were determined by flow cytometry. Antigen-specific CD8 T cell immunity was analyzed through stimulating PBMCs with CMV-EBV-Flu (CEF) viral peptide pool and subsequent intracellular IFNγ staining. Both peripheral CD8 and CD4 T cells from GM treated patients had a shift pattern characterized by an enlarged effector T_M_ and a reduced central T_M_ cell population when compared to GM untreated group. An increase in the frequencies of TNFα^+^, IL-2^+^, and IL-17^+^ CD8 T_M_ cells was observed whereas only TNFα^+^CD4 T_M_ cells increased in GM treated patients. Moreover, GM treated patients contained more peripheral IFNγ-producing CD8 T cells specific to CEF viral peptides. Together, these results show a distinct T cell subset pattern and enhanced memory T cell immunity upon GM treatment, suggesting an immunoregulatory effect of TNF inhibitor Golimumab on peripheral memory T cell responses.

## Introduction

Rheumatoid arthritis (RA) is an inflammatory joint disease that is characterized by a chronic inflammatory reaction in the synovium of joints with sustained release of inflammatory cytokines (1, 2). Among them, tumor necrosis factor α (TNFα) has been postulated to be a main cytokine in RA pathogenesis that is responsible for the clinical manifestations of the disease (3). It is produced mainly by monocytes, macrophages and to a lesser degree by T cells (3). TNFα can induce the production of other proinflammatory cytokines, such as IL-1 and IL-6, to exaggerate the inflammation. It is also responsible for the production and release of chemokines by monocytes and macrophages that attract leukocytes from the blood into the inflamed tissues. Moreover, over-production of TNFα leads to the destruction of the underlying articular cartilage and subchondral bone through induction of proteolytic and metalloproteinase enzymes production by activated macrophages and fibroblasts, and initiate osteoclast desorption (4). Along with the development and clinical application of first biological agent Infliximab, TNFα inhibitors (TNFIs) become a major advancement in the treatment of RA (5). They are endowed with profound immunoregulatory effects and possess high clinical efficacy in ameliorating clinical symptoms as well as retarding tissue damages particularly when used in combination with methotrexate (MTX) (5, 6). Reports from clinical investigations, on the other hand, reveal the regulatory roles of TNFIs on T cell functionality. Combined treatment of Etanercept and MTX was reported to reverse Th1/Th2 and Th17/regulatory T cells imbalance in RA patients, which were strongly associated with the amelioration of RA activity (7). However, no studies to date have investigated the functionality of memory T cells (T_M_) cells during TNFα blockade in RA.

T_M_ cells are main components to provide immune protection in recall immune responses such as re-infection. They are characterized by longer survival, stronger and faster recall responses upon secondary challenge (8, 9). T_**M**_ cells can be subgrouped into central and effector memory T cells based on their homing patterns, proliferative capacity as well as immune effects (10). Central memory T cells (T_CM_), which express lymph node homing receptor CCR7, mainly reside in secondary lymphoid organs with limited effector functions. CCR7-null effector memory T cells (T_EM_), on the other hand, circulate between blood, lymphoid organs and tissues. They exert immunosurveillance with high capacity to secrete huge amounts of effector cytokines more rapidly (10, 11). The maintenance, function and distribution of T_M_ cells are affected by many factors including inflammatory cytokines (12, 13). Disturbances in homeostasis and differentiation of T_M_ cells have been reported in early RA as well (14). CD4 T_M_ cells from untreated RA patients manifest intrinsic abnormalities in differentiating into cytokine producing effector cells (14). More importantly, the proportions of circulating CD4 and CD8 T_M_ cells correlate with level of the rheumatoid factor (IgM-RF), one of the important indicators for disease severity (15). However, little is known about the effects of TNFα inhibitor treatment on T_M_ cells in RA patients.

On the path of seeking optimal clinical outcomes in terms of efficacy and safety, the pipeline of TNFα antagonists has witnessed the emergence of more drugs, such as Golimumab (GM). GM is a human monoclonal antibody binding to both soluble and transmembrane forms of TNFα with high affinity and specificity. It neutralizes the bioactivity of TNFα by blocking the interaction between TNFα and its receptors (16). GM is one of the latest TNFα inhibitors evaluated for its efficacy in double-blinded random clinical trials (RCTs). When administered subcutaneously to RA patients in whom the response to methotrexate (MTX) is inadequate, GM plus MTX effectively reduce the signs and symptoms of the disease with less immunogenicity (17–20). In this study, relying on a clinical trial: “A phase 3, multicenter, randomized, double-blind placebo controlled study evaluating the efficacy and safety of Golimumab (GM) in the treatment of Chinese subjects with active rheumatoid arthritis despite methotrexate therapy” (21), we performed the analyses of the phenotypes and functions of T_**M**_ cells in two groups of RA patients either with conventional MTX treatment (GM untreated) for more than 6 months or MTX+GM treatment (GM treated) for a similar period as well as healthy controls (HCs). While memory T cells cytokine secreting function in GM untreated patients was inclined to normalize to HCs, CD4 and CD8 T_M_ cells from GM treated group showed enhanced cytokine production as well as an apparent memory subset bias with an enlarged T_EM_ compartment and a decreased T_CM_ when compared to GM untreated patients. Moreover, more responders to CMV-EBV-Flu (CEF) peptide pool stimulation as well as higher frequencies of FLU peptide-specific CD8 T cells were observed in the GM treated RA group.

## Material and Methods

### Subjects

Forty-nine RA patients fulfilling the 1987 diagnostic criteria of the American College of Rheumatology were recruited from the Department of Rheumatology and Immunology, in Shanghai Changzheng Hospital. Sixty-eight age and gender-matched healthy controls were included in the study. RA patients were divided into two groups: patients undergoing GM treatment was referred to as “**GM treated”** (*n* = 14). RA patients who didn't receive Golimumab, nor any other TNFα inhibitor as named “**GM untreated**” (*n* = 35), were served as treatment control group. GM treated patients participated in the clinical trial: “A phase 3, multicenter, randomized, double-blind placebo controlled study evaluating the efficacy and safety of Golimumab in the treatment of Chinese subjects with active Rheumatoid arthritis despite methotrexate therapy” (NCT01248780), and received 50 mg GM subcutaneously (s.c.) every 4 weeks for up to 48 weeks and a stable dose of MTX: 7.5–20 mg/week.

GM untreated patients received conventional disease modifying anti-rheumatic drug (DMARD) medications such as MTX (10 mg/week), Leflunomide (10 mg/day), together with hormones such as prednisone (10–15 mg/day) and non-steroidal anti-inflammatory drugs (NSAIDs) (1–2 pills/day). Further information regarding the patients' age, gender, disease activity and drug regime could be found in Table 1. Patients and HCs were matched for age and gender, Moreover, GM treated patients and untreated ones were matched for clinical duration. RA disease activity was assessed at the time of blood collection, using the Disease activity Score of 28 joint counts, levels of rheumatoid factor, erythrocyte sedimentation rate and the C-reactive protein (CRP) level.

**Table 1:** Characteristics of HC and RA patients.

	**Healthy controls**	**GM untreated RA**	**GM treated RA**
Number	65	35	14
Gender, Female/Male	30/4	31/4	13/1
Age, years	51.07 ± 11.03	53.15 ± 15.01	47.43 ± 11.35
**DIS ACTIVITY**			
RF, UI		143.2 ± 179.9	165.9 ±168.8
CRP, mg/dl		15.65 ± 24.42	8.4 ± 18.31
ESR, mm/hour		34.34 ± 14.98	25.43 ± 15.3
DAS28		4.51 ± 2.23	2.23 ± 0.86, *p* = 0.0055
Dis duration, years		8.36 ±9.37	6.16 ± 6.21
CT duration		/	10.33 ± 2.77 months
MTX use, %		74.28	100
MTX duration,months		≥6	10.33 ± 2.77

*RF indicates Rheumatoid Factor; CRP, C Reactive Protein; ESR, Erythrocyte Sedimentation Rate; DAS28, Disease Activity Score for 28 joints and CT duration: Clinical Trial duration*.

The study was approved by the Medical Ethics Committee of the Shanghai Chang Zheng Hospital and all experiments were performed according to the principles of the Declaration of Helsinki. Informed consent was assigned individually from all participants before enrollment.

### Reagents

PMA and Ionomycin were purchased from Sigma-Aldrich and used for non-specific stimulation and the positive controls. Mouse anti-human CD3-APC CY7 (clone SK7) and anti-IFNγ-APC were from BD Biosciences. Mouse anti-human CD8-PerCP (clone RPA-T8) was from BIOLEGEND. Mouse anti-human IL-2-allophycocyanin (clone 17-7029-71), anti-CD45RA-FITC (clone HI100), anti-CCR7-PE CY7 (clone 3D12), anti-TNFα-PE (clone MAB11) and anti-IL-17-PE (clone BL 168) were from eBioscience. Anti-human HLA-A2-PE (clone BB7.2) was from Abcam. CMV-EBV-Flu peptide pool was used for the study of antigen specific CD8 T cell responses as described previously (22). Peptides were synthesized by the GeneImmune (Suzhou, China), consisting of 23 HLA class I-restricted T cell epitopes from well-defined CMV, EBV and FLU viral proteins. The peptides were restricted by the most common HLA alleles including HLA-A1, A2, A3, A11, A24, A68, B7, B8, B27, B35, B44 (Supplementary Figure 4A) and pooled into 12 groups as indicated in Supplementary Figure 4B. Function-graded mouse anti-human CD28 and anti-CD49d antibodies were from BD Biosciences.

### Isolation of PBMCs

Whole blood was collected from patients and healthy volunteers in tubes containing 10 U/ml heparin. The blood was mixed with an equal volume of RPMI 1640 (Sigma) containing 2 mM Glutamine (Gibco, USA), 100 U/ml Penicillin and Streptomycin (Gibco). Peripheral blood mononuclear cells (PBMCs) were isolated by using Ficoll-hypaque density gradient centrifugation with LymphoprepTM (AXIS-SHIELD Poc AS, Norway) at 2,200 rpm for 20 min at 20°C. PBMCs were washed twice with RPMI 1640 medium by centrifuging at 1,500 rpm at 20°C for 5 min. Cells were resuspended in RPMI 1640 containing 10% fetal bovine serum (FBS) (Gibco), 2 mM Glutamine (Gibco), and 100 U/ml Penicillin and Streptomycin (Gibco) for further experiments, including FACS staining and peptide stimulation.

### HLA-A2 Allele Determination

Following PBMC separation, 0.1^*^10^6^ of cells were re-suspended in FACS buffer containing anti-human HLA-A2-PE antibody and incubated for 30 min at 4°C. Cells were then washed and re-suspended in PBS and immediately analyzed by CANTO II flow cytometer (BD Biosciences).

### CEF Peptide Stimulation

Briefly, 100 μl PBMCs (1 × 10^6^) were stimulated individually with 12 CEF peptide pools (Supplementary Figure 1A) at a final concentration of 2 μg/ml per peptide in the presence of anti-CD28 antibody (2 μg/ml), anti-CD49d antibody (4 μg/ml) and Golgi stop (1.5 μl/ml) (BD Bioscience). Stimulation with costimulatory antibodies and Golgi stop was treated as negative control. After 6 h incubation at 37 C in 5% CO_2_, cells were subjected to flow cytometric analysis.

### Flow Cytometry

T cell subsets were analyzed by flow cytometry with anti-CD3, anti-CD8, anti-CD45RA, anti-CCR7 antibodies. Anti-HLA-A2-PE antibody was used to specifically determine HLA-A2 allele. Briefly, 10^6^ PBMCs were stained with antibodies for 30 min at 4°C, washed twice with PBS and acquired by Canto II flow cytometer (BD Biosciences). For Intracellular cytokine staining, cells were stimulated with PMA (100 μg/ml) (Sigma-Aldrich), ionomycin (1 mg/ml) (Sigma-Aldrich) and Golgi stop (1.5 μl/ml) (BD biosciences) or CFE peptide pools for 6 h. Intracellular cytokine staining was performed according to the protocol of Cytofix/Cytoperm kit (BD Biosciences). After staining, cells were immediately acquired on a CANTO II flow cytometer (BD Biosciences). Cell doublets were excluded using forward light scatter-area vs. forward light scatter-height parameters. Data analysis was performed with FACS Diva software (BD Biosciences) or FlowJo 9.3.2 software (FLOWJO LLC, OR, USA).

### Statistical Analysis

The data were represented by mean ± S.E.M. Statistical analyses were performed with GraphPad Prism 5 software (GraphPad Software Inc., CA, USA). Nonparametric Mann-Whitney test was used for comparison between two groups. *P* < 0.05 was considered statistically significant.

## Results

### Demographic Indicators and Clinical Characteristics of RA Patients

The demographic indicators and main characteristics of RA patients as well as HCs in this study were summarized in Table 1. Patients and HCs were matched for age and gender. Moreover, both GM treated patients and untreated ones received MTX treatment for indicated period of time (>6 months, Table 1). GM treated group received additional GM treatment (10.33 ± 2.77 months, Table 1). They were matched for clinical duration, RF titers, ESR and CRP levels. Only DAS 28 was increased in the untreated group (*p* = 0.0055) indicating a moderate disease activity. However, the absence of significant differences in the inflammatory markers represented by ESR and CRP between the GM untreated and GM treated group indicate that the inflammatory milieu is comparable between both groups.

### Distinct Patterns of CD8 and CD4 T Cell Subsets Upon Golimumab Treatment

**Figure 1:**
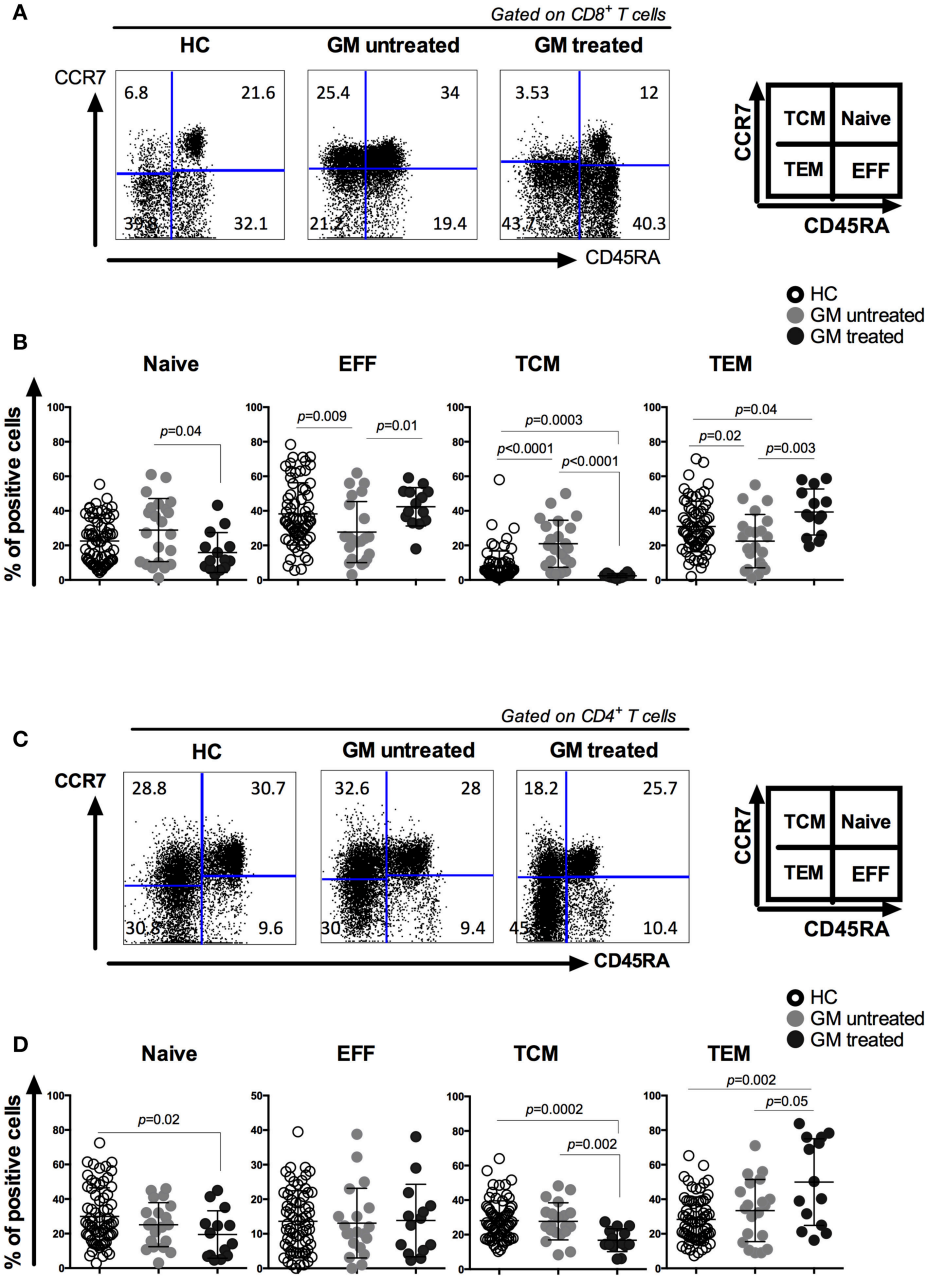
CD8 and CD4 T cells from GM treated patients have increased effector memory T cell compartment associated with a decreased central memory one as compared to both GM untreated patients and HC group. Representatives of CD8 **(A)** and CD4 **(C)** T cell subsets from one GM treated RA patient, one GM untreated patient and a gender- and age-matched control individual. CD8 and CD4 T cells were subgrouped into naïve (T_N_), effector (T_E_), central memory (T_CM_) and effector memory (T_EM_) subpopulations by using anti-CD45RA and anti-CCR7 antibodies. Pooled data of T_N_, T_E_, T_CM_, and T_EM_ subpopulations in CD8 **(B)** and CD4 **(D)** T cells from GM untreated patients (*n* = 23), GM treated patients (*n* = 14) and control individuals (*n* = 68). Horizontal lines indicate mean values.

The effect of GM treatment on CD4/CD8 ratio was first assessed and compared between GM treated, GM untreated RA patients and HCs (Supplementary Figure 1). It is noteworthy that the ratio was comparable between the three groups and that no significant differences were observed. Next, we analyzed the effects of GM treatment on the frequencies of CD8 and CD4 T cell subsets, including naïve (T_N_), effector (T_E_), central memory (T_CM_) and effector memory (T_EM_) subpopulations based on CCR7 and CD45RA expression (Figures 1A,C). Our results revealed that less CD8 T_N_ cells and more CD8 T_E_ cells were observed in GM treated RA patients as compared to untreated ones (*p* = 0.04 and *p* = 0.01, respectively). Higher frequencies of T_EM_ associated with a dramatic decrease in T_CM_ were observed in GM treated patients when compared to GM untreated patients (*p* = 0.003, *p* < 0.0001) or HC (*p* = 0.04, *p* = 0.0003) (Figure 1B).

Memory CD4 T cell compartment has also shown the same shift from T_CM_ to T_EM_ in GM treated patients with higher frequencies of T_EM_ and lower frequencies of T_CM_ population as compared to GM untreated group (*p* = *0.05, p* = *0.002*) or HC (*p* = *0.002, p* = *0.002*). However, no differences were observed in the frequencies of T_N_ and T_E_ subsets between GM treated and untreated group (Figure 1D). These results indicate that GM treatment mainly modulate the homeostasis of two T_M_ subsets rather than T_N_ or T_E_ subpopulations.

### More TNF-α, IL-2, and IL-17-Producing CD8 T_M_ cells and TNF-α Producing CD4 T_M_ Cells in GM Treated Patients

**Figure 2:**
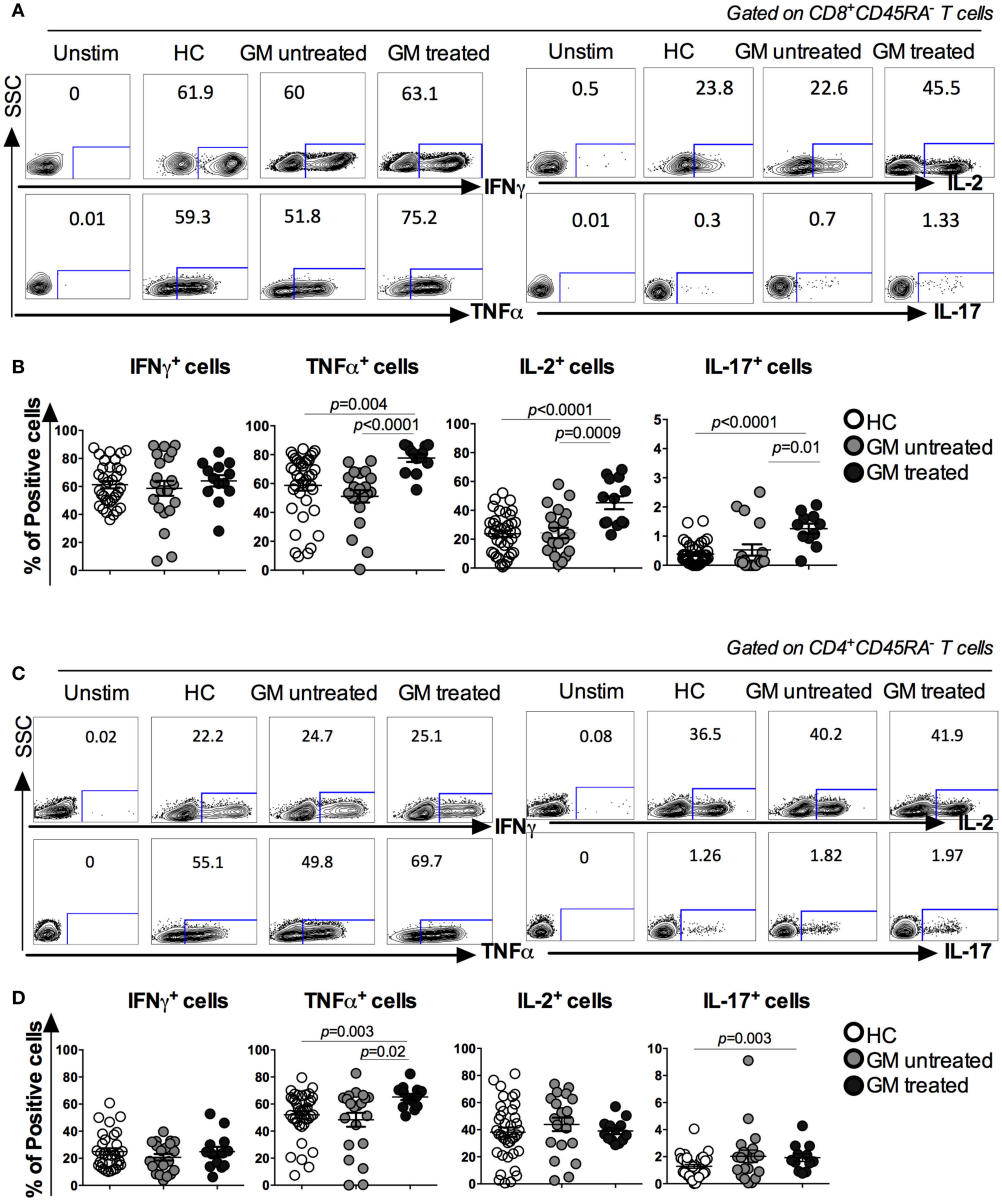
Increased cytokine production in CD8^+^CD45RA^−^ and CD4^+^CD45RA^−^ T cells in GM treated patients. Freshly isolated PBMCs were stimulated with PMA/Iono for 6 h and IFNγ, TNFα, IL-2, and IL-17 production were detected by intracellular cytokine staining. **(A,C)** representatives of ICS analysis of CD8 and CD4 T cells from HC, GM untreated and GM treated RA patients. **(B)** Statistical analysis of IFNγ, TNFα, IL-2, and IL-17 secreting CD8 T_M_ cells in GM untreated patients (*n* = 21), GM treated patients (*n* = 14) and control individuals (*n* = 68). **(D)** Statistical analysis of IFNγ, TNFα, IL-2, and IL-17 secreting CD4 T_M_ cells from GM untreated patients (*n* = 21), GM treated patients (*n* = 14) and control individuals (*n* = 68).

The function of peripheral CD8 and CD4 T_M_ cells was further assessed based on the capacity of cytokine production among three groups. Freshly isolated PBMCs were stimulated *in vitro* with PMA/Ionomycin. Cytokine expression in CD8^+^CD45RA^−^ and CD4^+^CD45RA^−^ T_M_ cells, including IFNγ, TNFα, IL-2, and IL-17, was analyzed (Figures 2A,C). Results indicated that CD8 T_M_ cells from GM treated RA patients exhibited a significant increase in the percentages of TNFα, IL-2, and IL-17 production when compared to both GM untreated (*p* < *0.0001, p* = *0.0009, p* = *0.01* respectively) or HC groups (*p* = *0.004, p* < *0.0001, p* < *0.0001*, respectively). However, no difference was observed in IFNγ-secreting CD8 T_M_ cells among three groups (Figure 2B). Similar to CD8 T_M_ cells, CD4 T_M_ cells from GM treated RA patients exhibited a significant increase in TNFα expression when compared to GM untreated (*p* = 0.02) or HC (*p* = 0.003) groups (Figure 2D). No difference was observed in the percentages of IFNγ^+^ and IL-2^+^ CD4 T_M_ cells among three groups. Taken together, both CD4 and CD8 T_M_ cells exhibit enhanced functionality reflected by significant increase in some key cytokines expression after TNFα blockade in RA patients.

Moreover, analysis of cytokine expression within CD8 and CD4 T_M_ subsets (T_CM_ and T_EM_) (Supplementary Figures 2, 3) revealed an enhanced expression of TNFα and IL-2 in CD8 T_CM_ in GM treated as compared to GM untreated (*p* = 0.0004 and *p* = 0.0001, respectively) or HC (*p* = 0.0001 and *p* = 0.0024, respectively). Also, TNFα in CD4 T_CM_ and T_EM_ increased in GM treated as compared to HC (*p* < 0.0001 and *p* = 0.0012, respectively). Of interest, this enhanced profile was associated with a restored expression of IFNγ from CD4 T_EM_ and terminally differentiated CD8 T_E_ and CD4 T_E_ cells that had increased frequencies in GM treated as compared to GM untreated (*p* = 0.0039, *p* = 0.0087 and *p* < 0.0001, respectively). Frequencies of IL-2^+^ and TNFα^+^ cells within CD8 T_EM_ increased as well in GM treated as compared to GM untreated to be normalized with HC group (*p* = 0.0001 and *p* = 0.0001). Overall, these findings highlight an enhanced and/or restored profile of cytokine responses of memory T cells upon Golimumab treatment.

### Stronger Viral-Specific CD8 T Cell Responses in GM Treated RA Patients

Considering the alterations of cell subsets and cytokine production of CD8 T_M_ cells in GM treated RA patients, we next examined the virus-specific CD8 T cell responses using *in vitro* stimulation of viral peptides (22). 23 HLA class I-restricted T cell peptides from CMV, EBV and FLU viruses (Supplementary Figure 4A) were synthesized and pooled into 12 peptide pools (Supplementary Figures 4B,C). 10 pools from A to J containing five or four peptides each, one FLU pool containing 7 FLU peptides and one EBV pool containing 13 peptides from EBV virus (Supplementary Figure 4B). PBMCs from GM treated (*n* = 14), GM untreated (*n* = 22) and HCs (*n* = 43) were stimulated with individual peptide pools separately and IFNγ-expressing CD8 T cells were determined after stimulation. For instance, when patients responded to both pool A and J determined by IFNγ production, they were defined as responders to HLA-A2 restricted FLU peptide-5, GILGFVFTL (Supplementary Figures 4B,C).

**Figure 3:**
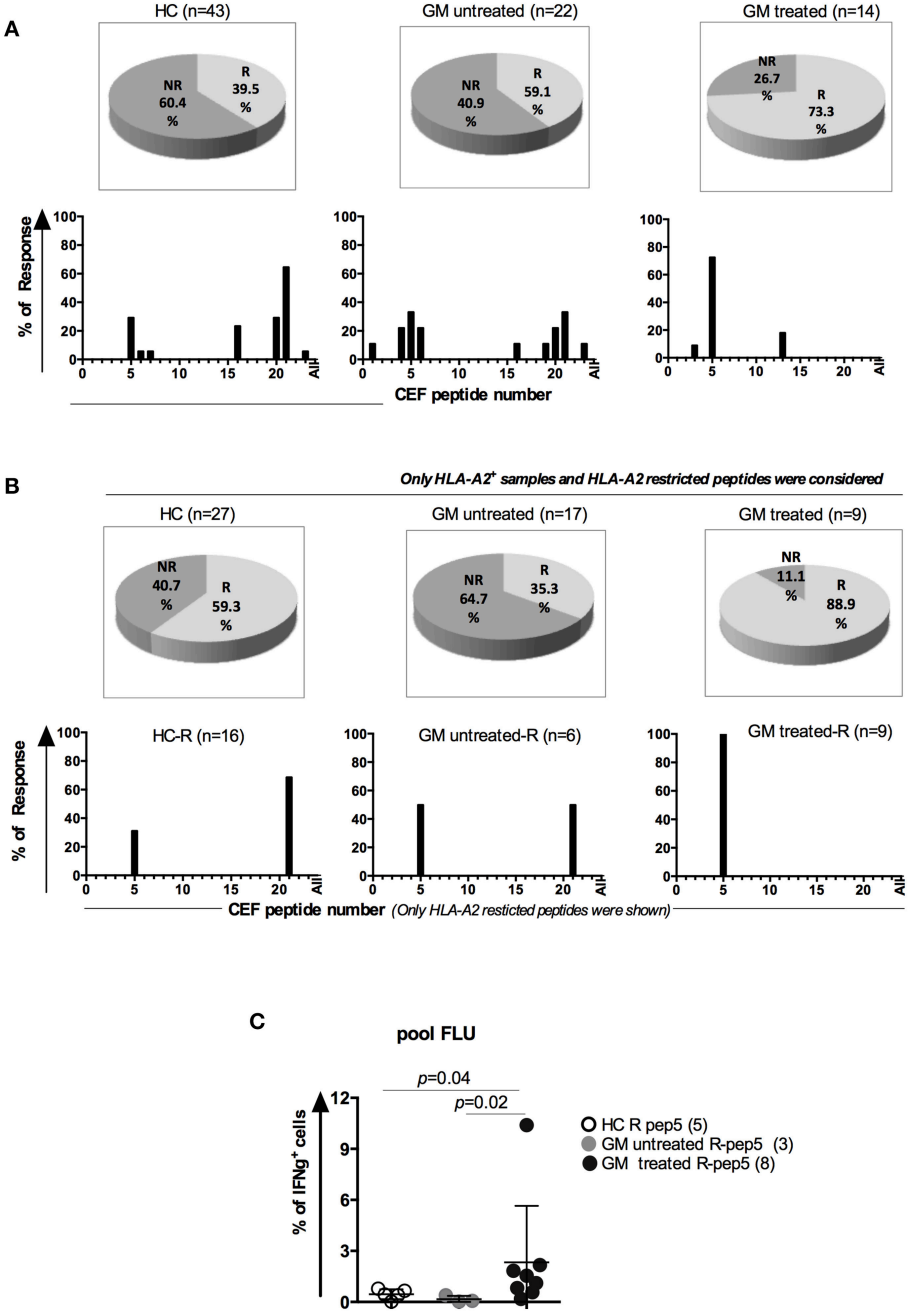
Enhanced antigen-specific memory T cell responses in GM treated RA patients. **(A)** Upper panel**:** Percentages of responders (R) and non-responders to CEF peptide stimulation in three groups. Lower panel: peptide distribution in responders **(B)** Upper panel**:** Percentages of HLA-A2^+^ R and NR to HLA-A2 restricted peptides in three groups; Lower panel: peptide distribution in HLA-A2^+^ responders. **(C)** Frequencies of IFNγ^+^ CD8^+^ T cells in responders to the peptide GILGFVFTL (FLU-A2) in GM treated patients (*n* = 8), GM untreated patients (*n* = 3) and HCs (*n* = 5).

More responders were observed in GM treated group (73.3%) than GM untreated (59.1%) or HC (39.5%) groups (Figure 3A). When we compared the responders to HLA-A2 restricted peptides in HLA-A2 population among three groups, the difference was more dramatic. 88.9% of GM treated RA patients were reactive to HLA-A2-restricted peptides whereas only 35.5 % in GM untreated RA patients and 59.3% in HC controls (Figure 3B). Among all viral peptides, HLA-A2 restricted Flu peptide-5 (GILGFVFTL) was most frequently responded by GM treated (100%), GM untreated (50%) and HC (31.25%) individuals (Figure 3B).

We further compared the frequencies of IFNγ^+^ CD8^+^ T cells specific to pooled FLU peptides among HLA-A2^+^ responders from three groups. Our results indicated that GM treated patients had more IFNγ^+^CD8^+^ T cells after stimulation with Flu peptide pool (2.3 ± 3.32%) when compared to GM untreated responders (0.16 ± 0.2%, *p* = 0.02) or HCs (0.45 ± 0.29%, p = 0.04), (Figure 3C). From above results, we conclude that antigen-specific CD8 T cells display enhanced functionality upon GM treatment as well.

## Discussion

In the present study, we have elucidated the impact of a newly developed anti-TNFα human antibody, Golimumab on circulating T_M_ cells in RA patients. We found that T_M_ cells from GM treated RA patients were characterized by distinct subset pattern and enhanced functional profiles when compared to GM untreated RA patients.

It has been demonstrated previously that a skewed differentiation of circulating CD8 T cells is considered as a main feature of RA characterized by an increase in CD45RA^−^CD62L^+^ CD8 T_CM_ cells and a concomitant decrease in CD45RA^+^CD62L^−^ CD8 T_E_ cells (23). In line with this study, our results displayed the same pattern in the GM untreated cohort where the compartment of CD8 T_CM_ cells was significantly enlarged whereas CD8 T_EM_ and T_E_ cells were retracted as compared to HC group. However, GM therapy exhibited a reversed pattern as compared to GM untreated patients. Frequencies of CD8 T_CM_ decreased significantly and those of CD8 T_E_ and T_EM_ subsets increased, which is more similar to the profiles of HC group. This observation is present as well in CD4 T cell compartment, suggesting that GM treatment is commensurate with accentuated recovery of normal T cell homeostasis.

We further evaluated the impact of GM on the function of T_M_ cells in terms of cytokine production including IFNγ, TNFα, IL-2, and IL-17. First, we noticed that GM untreated patients and healthy controls had a similar cytokine expression profile, which might reflect a normalization effect exerted by MTX therapy in the GM untreated group (MTX use 74.28%, Table 1); In fact, previous studies have shown a decline in CD8^+^IFNγ^+^ cells (24) and a reduction in circulating Th17 subsets after MTX treatment (25). Moreover, long-term therapy with MTX in combination with low dose corticosteroids for RA influenced the predominance of type 1 cytokines toward normalization of the cytokine balance in both CD4^+^ and CD8^+^ T lymphocytes (26). Of interest, the GM treated RA patients group displayed rather increased percentages of IL-2 and IL-17-producing CD8 T_M_ cells when compared to GM untreated or HC groups. This enhancement could be speculated in part by the inhibitory effects mediated by TNFα where TNFα has been reported to exert an inhibitory effect on T cell function *in vitro* and *in vivo* (27). The phenotypes observed in GM treated patients help understand the inhibitory effects of TNFα in human, which might represent partially a negative feedback loop that attempts to limit the intensity and/or duration of T_M_ responses. Although, no effect was observed on IFNγ production in total T_M_ in both CD4 and CD8 populations upon GM treatment in RA patients, we have observed increased proportions of this cytokine in CD4 T_EM_ in the GM treated group as compared with the GM untreated and consequently normalization with HC. This restored profile was observed as well in CD4 T_E_ and CD8 T_E_ subpopulations. IFNγ production by T cells in RA patients has been demonstrated to increase after TNF inhibition therapy (28, 29), but data of memory T cells are lacking. The most dramatic and common alteration of cytokine production in CD4 and CD8 T_M_ cells upon GM treatment lies in the increase of TNFα production as compared to the control groups. This probably reflects a feedback mechanism by which T_M_ cells tend to compensate for the overall reduction of TNFα when neutralized by TNFα antibodies.

Consistent with our findings, Notley et al. reported that in collagen-induced arthritis mice model, TNFα blockade using TNFR-Fc fusion protein or anti-TNF monoclonal antibodies reduced arthritis severity but, unexpectedly, expanded populations of Th1 and Th17 cells (30). It was found that TNFα exerted a negative feedback on IFN-γ and IL-17 production through down-regulation of the common IL-12/IL-23 p40 unit, which is essential in the differentiation program of Th1 and Th17 cells. Moreover, anti-TNFα therapy prevented migration of T cells to the joints. Aerts et al. also found that anti-TNF therapy in RA patients increased the number of peripheral Th17 and Th1 cells as well as reduced the expression of CCR6 in anti-TNFα induced remission (31). Indeed, the effects of *in vivo* TNFα blockade on T cell accumulation in the synovium (32) due to the altered expression of homing receptors (28, 33, 34) has been well-established, and might explain the profile observed in our study.

Differential effects of different TNFα inhibitors on T cell function were only reported in ankylosing spondylitis. Treatment with the chimeric anti-TNFα monoclonal antibody Infliximab leads to a significant decrease in IFNγ and TNFα production by CD4 and CD8 T cells and was associated with a significant reduction in the number of antigen specific IFNγ^+^ and TNFα^+^ CD8 T cells (35). However, treatment with TNF receptor-Fc fusion protein (TNFR:Fc) Etanercept was rather associated with up-regulation of TNFα and IFNγ production by T cells accompanied with a good clinical outcome (36). To date, there are no similar comparative reports in RA, which needs to be investigated in the future. Comparing the effects of different TNFIs on T cell functionality might facilitate the determination of TNFIs for individualized application against RA.

Not only the increased TNFα, IL-2, and IL-17 production in CD8 T_M_ cells from GM treated group, we also observed more antigen-specific responders in GM treated RA patients following stimulation with viral peptides. A significant increase in the frequency of FLU-specific IFNγ production in CD8 T cells was noted in HLA-A2^+^ GM treated RA patients as compared to GM untreated patients and HC group. Actually, it was reported that GM treatment exhibited lower incidence of infections with a risk ratio (RR, 95% CI) of 0.99 (0.79–1.24) as compared to infliximab, another TNFα blockers with a RR of 1.23 (0.94–1.61) (37).

Several points illuminate the potential significance of our results. First, It is very interesting that after GM treatment for several months, RA patients displayed increased memory T cells responses including more T_EM_ cells and antigen-specific T cell cytokine production, strongly suggesting that TNFα blockade enhances the antigen-specific T cells responses. The increase in antigen-specific T cell response observed in our study might be an indicator of viral infection or reflect the enhanced ability of the host to viral infection. Although the exact consequence and mechanisms need to be further clarified, clinical application of anti-TNFα blockade provides an opportunity for us to elucidate the immunoregulatory properties of TNFα on human memory T cell responses. Our results might highlight another evidence for the benefit of TNFα blockade treatment for RA patients.

## Ethics Statement

The study was approved by the Medical Ethics Committee of the Shanghai Chang Zheng Hospital and all experiments were performed according to the principles of the Declaration of Helsinki. Informed consent was assigned individually from all participants before enrollment.

## Author Contributions

HS, YW, AK, BJ and HX designed the experiments. AK and BJ conducted the experiments. AK, YW, HS and HX analyzed the data. LZ, YJ, JY, JS and HX collected the samples and clinical data. PJ and SW contributed reagents, materials and analysis tools. AK, HS, YW and HX wrote the manuscript.

### Conflict of Interest Statement

The authors declare that the research was conducted in the absence of any commercial or financial relationships that could be construed as a potential conflict of interest.

## Supplementary Material

Supplementary Figure 1 CD4/CD8 T cell ratio in GM treated as compared to both GM untreated patients and HC group.

Supplementary Figure 2 Statistical analysis of IFNγ, TNFα, IL-2, and IL-17 secreting CD8 T cells from: T_N_, T_CM_. T_EM_, and T_E_ subsets in GM untreated patients (*n* = 15), GM treated patients (*n* = 14) and control individuals (*n* = 28).

Supplementary Figure 3 Statistical analysis of IFNγ, TNFα, IL-2, and IL-17 secreting CD4 T cells from: T_N_, T_CM_. T_EM_, and T_E_ subsets in GM untreated patients (*n* = 15), GM treated patients (*n* = 14) and control individuals (*n* = 28).

Supplementary Figure 4 **(A)** List of HLA Class I restricted 23 peptides from CMV, EBV, and Flu virus. The viral antigen, the amino acid sequence and HLA class I alleles of each epitope were listed. **(B)** Peptides were pooled into 12 groups (Pool A to pool J, FLU pool and EBV pool) for peptide stimulation assay. Those who reacted to both pool A and pool J were responded to Pep-05 (FLU peptide GILGFTFVL) with HLA-A2 restriction. **(C)** Representative of IFN-γ ICS analysis responding to pool A, pool J and FLU in one healthy control individual, one GM untreated patient and one GM treated patients.
