# Supplementary figures and images for: Restored and Enhanced Memory T Cell Immunity in Rheumatoid Arthritis After TNFα Blocker Treatment
Source: Front Immunol. 2019 Apr 24;10:887. doi: 10.3389/fimmu.2019.00887 (PMC6499160; doi:10.3389/fimmu.2019.00887)

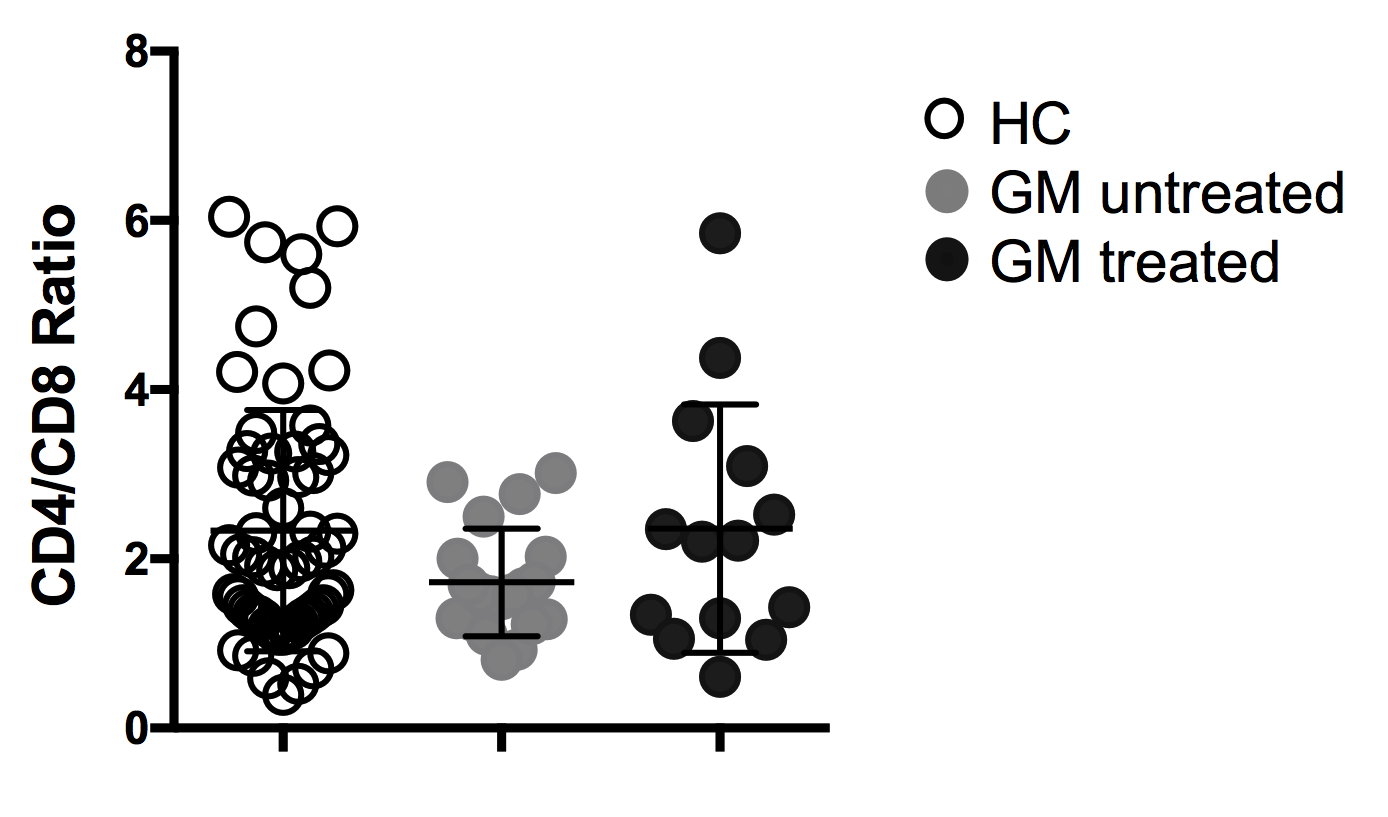

Supplement: Supplementary Figure 1 — CD4/CD8 T cell ratio in GM treated as compared to both GM untreated patients and HC group. [file Image_1.TIFF]

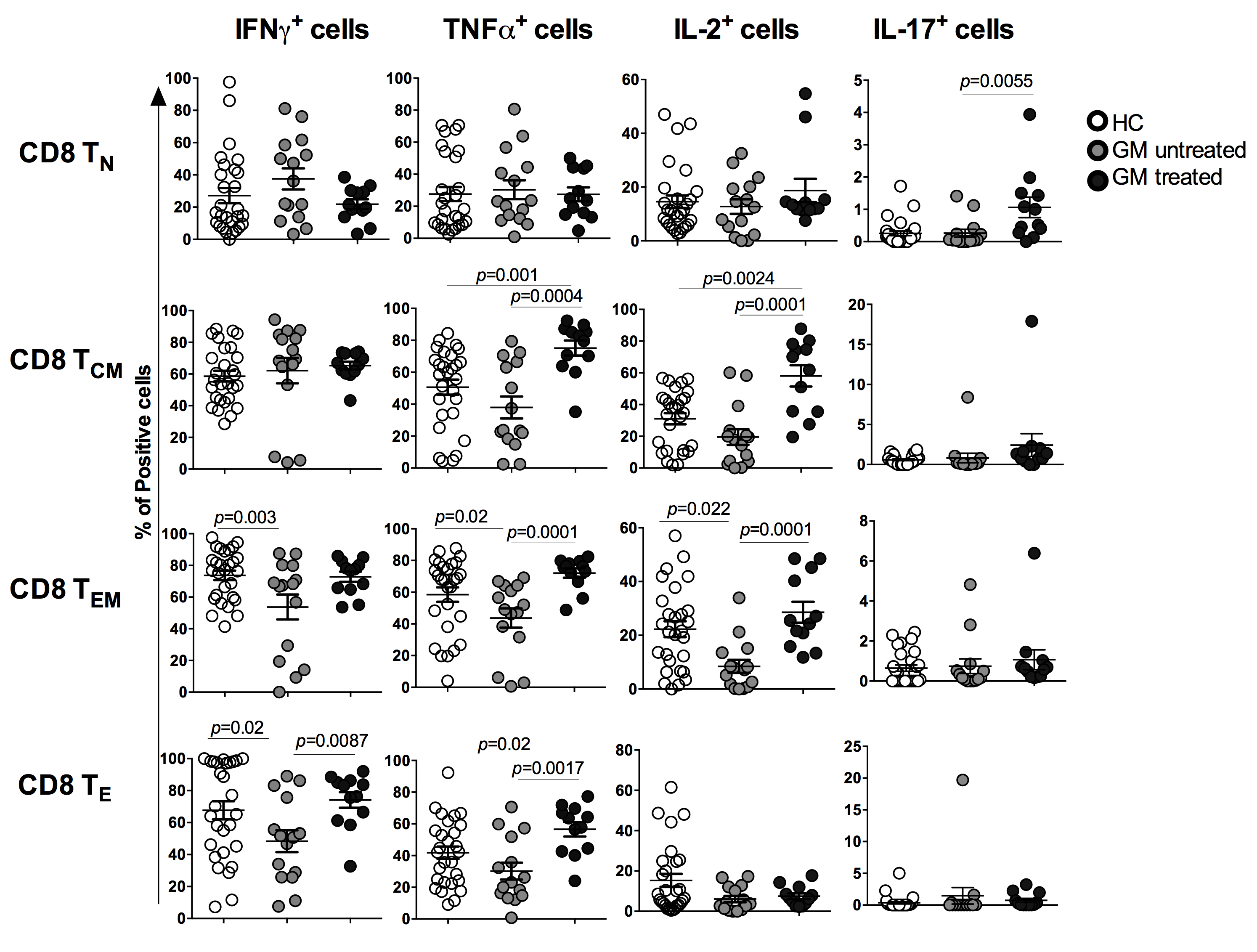

Supplement: Supplementary Figure 2 — Statistical analysis of IFNγ, TNFα, IL-2, and IL-17 secreting CD8 T cells from: TN, TCM. TEM, and TE subsets in GM untreated patients (n = 15), GM treated patients (n = 14) and control individuals (n = 28). [file Image_2.TIFF]

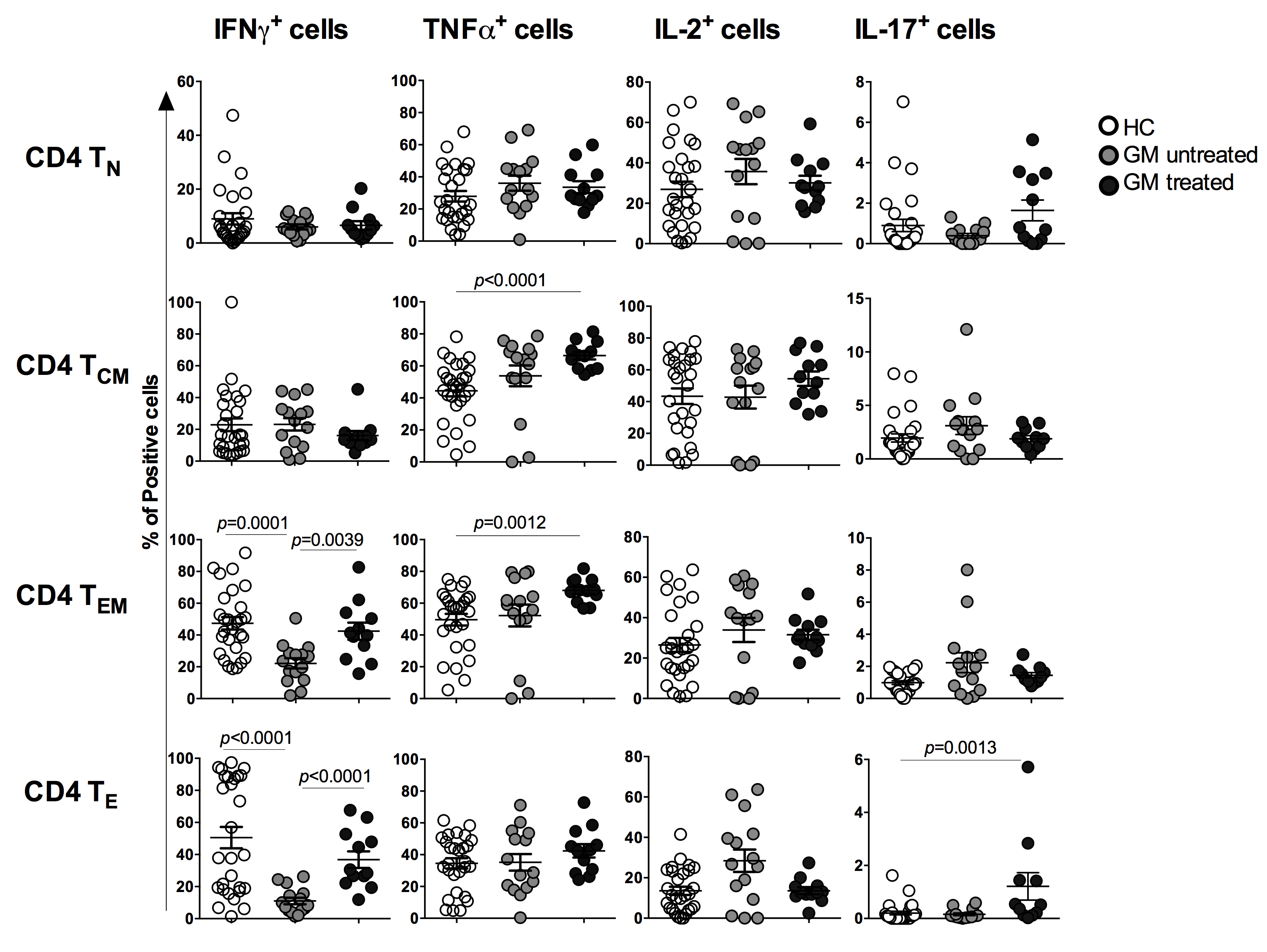

Supplement: Supplementary Figure 3 — Statistical analysis of IFNγ, TNFα, IL-2, and IL-17 secreting CD4 T cells from: TN, TCM. TEM, and TE subsets in GM untreated patients (n = 15), GM treated patients (n = 14) and control individuals (n = 28). [file Image_3.TIFF]

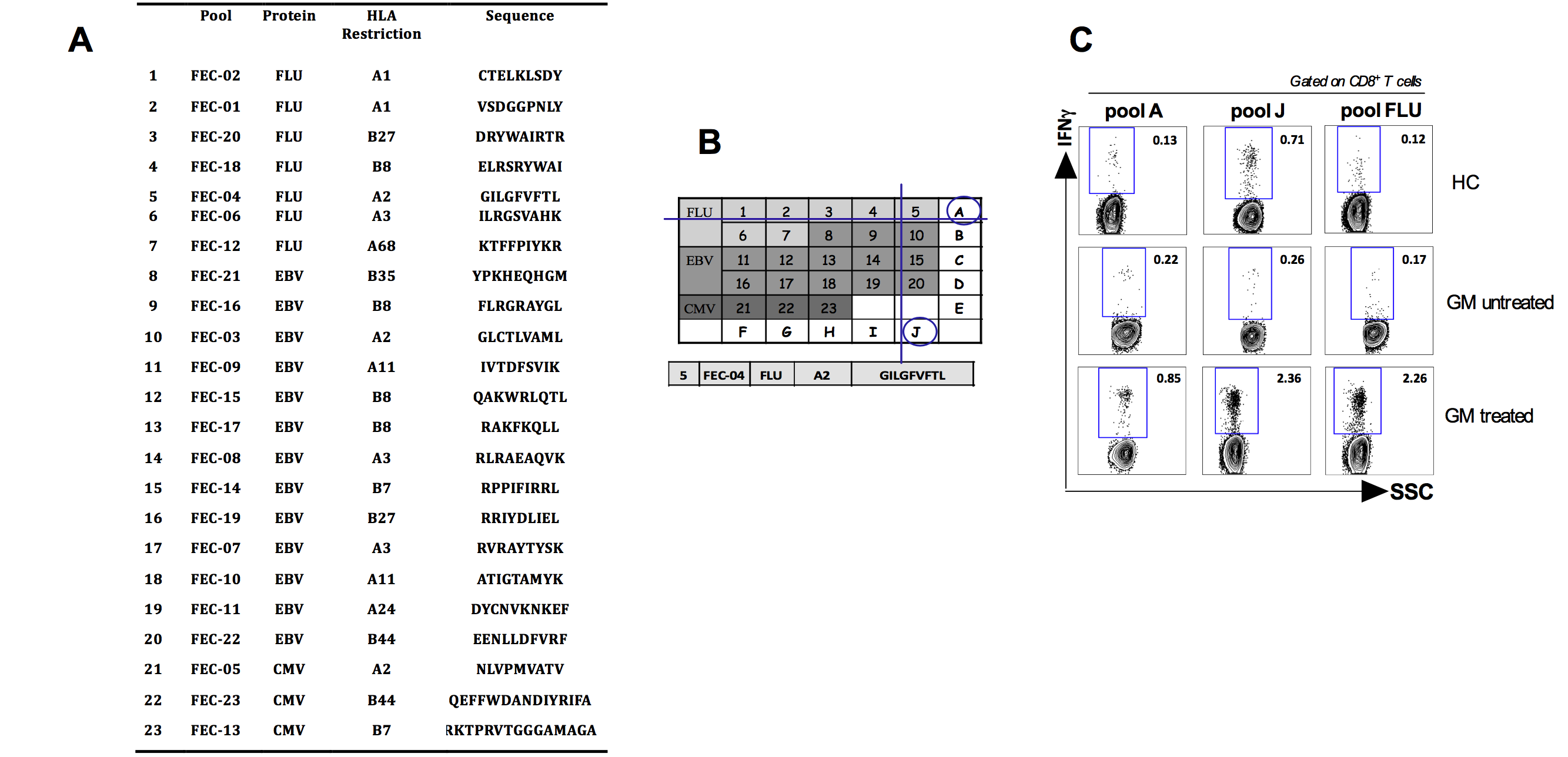

Supplement: Supplementary Figure 4 — (A) List of HLA Class I restricted 23 peptides from CMV, EBV, and Flu virus. The viral antigen, the amino acid sequence and HLA class I alleles of each epitope were listed. (B) Peptides were pooled into 12 groups (Pool A to pool J, FLU pool and EBV pool) for peptide stimulation assay. Those who reacted to both pool A and pool J were responded to Pep-05 (FLU peptide GILGFTFVL) with HLA-A2 restriction. (C) Representative of IFN-γ ICS analysis responding to pool A, pool J and FLU in one healthy control individual, one GM untreated patient and one GM treated patients. [file Image_4.TIFF]
